# Surveillance for Seasonal Influenza Virus Prevalence in Hospitalized Children with Lower Respiratory Tract Infection in Guangzhou, China during the Post-Pandemic Era

**DOI:** 10.1371/journal.pone.0120983

**Published:** 2015-04-13

**Authors:** Wen Da Guan, Xiao Yan Gong, Chris Ka Pun Mok, Ting Ting Chen, Shi Guan Wu, Si Hua Pan, Benjamin John Cowling, Zi Feng Yang, De Hui Chen

**Affiliations:** 1 State Key Laboratory of Respiratory Disease, National Clinical Research Center for Respiratory Disease, First Affiliated Hospital of Guangzhou Medical University, Guangzhou, China; 2 Department of Pediatric, First Affiliated Hospital of Guangzhou Medical University, Guangzhou, China; 3 Centre of Influenza Research, School of Public Health, HKU Li Ka Shing Faculty of Medicine, The University of Hong Kong, Hong Kong, China; 4 HKU-Pasteur Research Pole, School of Public Health, HKU Li Ka Shing Faculty of Medicine, The University of Hong Kong, Hong Kong, China; 5 Division of Epidemiology and Biostatistics, School of Public Health, The University of Hong Kong, Hong Kong, China; US Food and Drug Administration, UNITED STATES

## Abstract

**Background:**

Influenza A(H1N1)pdm09, A(H3N2) and B viruses have co-circulated in the human population since the swine-origin human H1N1 pandemic in 2009. While infections of these subtypes generally cause mild illnesses, lower respiratory tract infection (LRTI) occurs in a portion of children and required hospitalization. The aim of our study was to estimate the prevalence of these three subtypes and compare the clinical manifestations in hospitalized children with LRTI in Guangzhou, China during the post-pandemic period.

**Methods:**

Children hospitalized with LRTI from January 2010 to December 2012 were tested for influenza A/B virus infection from their throat swab specimens using real-time PCR and the clinical features of the positive cases were analyzed.

**Results:**

Of 3637 hospitalized children, 216 (5.9%) were identified as influenza A or B positive. Infection of influenza virus peaked around March in Guangzhou each year from 2010 to 2012, and there were distinct epidemics of each subtype. Influenza A(H3N2) infection was more frequently detected than A(H1N1)pdm09 and B, overall. The mean age of children with influenza A virus (H1N1/H3N2) infection was younger than those with influenza B (34.4 months/32.5 months versus 45 months old; p<0.005). Co-infections of influenza A/ B with mycoplasma pneumoniae were found in 44/216 (20.3%) children.

**Conclusions:**

This study contributes the understanding to the prevalence of seasonal influenza viruses in hospitalized children with LRTI in Guangzhou, China during the post pandemic period. High rate of mycoplasma pneumoniae co-infection with influenza viruses might contribute to severe disease in the hospitalized children.

## Introduction

In March and April of 2009, cases of human infection with the swine origin human H1N1 influenza A virus were first detected in North America [1,2]. During the early outbreak, patients with confirmed influenza A(H1N1)pdm09 tended to present with relatively severe clinical symptoms such as respiratory failure and multiple organ damage [2–4]. However, a majority of cases were subsequently found to show a mild course of disease similar to the seasonal influenza virus infection [5]. In August 2010, the World Health Organization declared the start of the post-pandemic phase [6]. Infection with the influenza A(H1N1)pdm09 virus was first reported in China on May 11, 2009 and increased community transmission of this subtype was observed subsequently with a peak in activity in November 2009 [4].

It has been known that influenza A(H1N1)pdm09, A(H3N2) and B viruses have continued to circulate in populations after the pandemic period. A number of reports have documented the burden of influenza in the post-pandemic period from different countries [7–10]. However, there is very limited data on the contribution of influenza virus to the burden of hospitalization in children in China. Report showed that approximately 70% of hospitalized patients with influenza infection in severe acute respiratory infection (SARI) cases were children under 5 years old [8]. Children with influenza infection usually present with upper respiratory tract infection symptoms. However, some of them developed lower respiratory tract infection (LRTI) such as bronchitis or pneumonia, resulting in the need for hospitalization. During the pandemic period, we have established a surveillance system to monitor the burden of influenza in Guangzhou, which is the largest metropolitan city in Guangdong province. A three-year surveillance study from January 2010 to December 2012 was carried out in the city of Guangzhou to estimate the prevalence of influenza virus infection in the hospitalized children with LRTI in Guangzhou during the post pandemic period.

## Materials and Methods

### Subjects

A retrospective study was conducted in hospitalized children with LRTI at the First Affiliated Hospital of Guangzhou Medical University, which is a large tertiary referral hospital in Guangzhou. The inclusion criteria for the study were clinical diagnosis of LRTI defined as the presence of at least one respiratory symptom (cough, sputum production, dyspnea, tachypnea, pleuritic pain) along with at least one finding during auscultation (rales, crepitation), or one sign of infection (core body temperature>38.0°C, shivering, leukocyte count >10×10^9^/L or<4×10^9^/L cells) independent of antibiotic pretreatment.

Throat swab samples from the patients were tested for influenza A H1N1pdm09, seasonal H3N2 and influenza B virus infection using real-time RT-PCR as described previously [11]. The clinical features and other factors among the three infection groups were compared. This study was approved by the ethics committee of the First Affiliated Hospital of Guangzhou Medical University which was waived the need for written consent since virologic testing was a routine diagnostic procedure and patient information saved at the study database was delinked from individual patient identifiers. The infection of mycoplasma pneumoniae was determined by an indirect immunofluorescent assay kit (PNEUMOSLIDE IgM, Vircell) for the simultaneous diagnosis in human serum of IgM antibodies. The presence of bacteria from throat swab, induced sputum or bronchoalveolar fluid were identified by VITEK 2 Compact system (BioMe´rieux, France).

### Statistical Analysis

GraphPad Prism 5 was employed for statistical analysis. Quantitative data are presented as the mean±standards deviation (SD) or median values with range. Normally distributed data were compared by analysis of variance(ANOVA); Otherwise, the non-parametric Kruskal-Wallis test was used. The categorical variables are reported as frequencies and percentages following comparison with Fisher’s exact or a chi-square test. All hypothesis testing was two-sided; Levels of significance were set at a p value <0.05.

## Results

**Fig 1:**
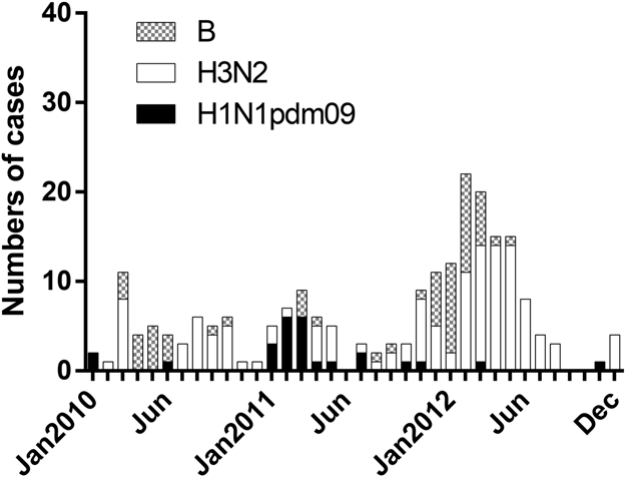
Number of influenza positive by subtype and by month of illness onset among the hospitalized children with lower respiratory tract infection (N = 3637), Guangzhou, China, January, 2010 to December, 2012.

**Fig 2:**
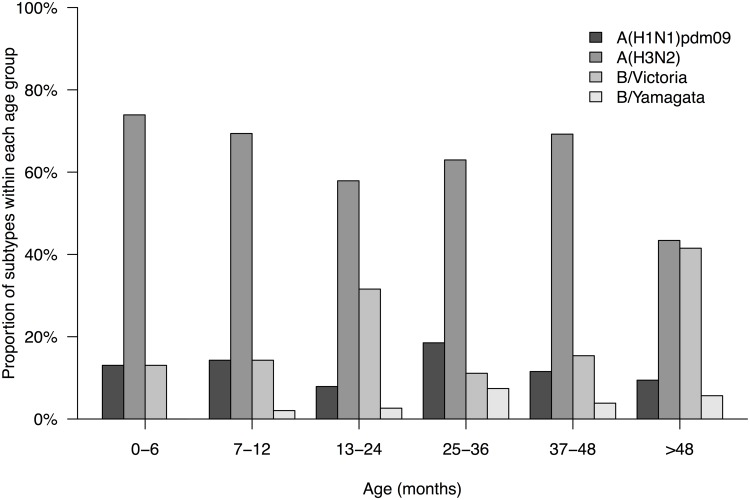
Estimated hospitalization rates (%) attributable to influenza infection by age group and by type/subtype in Guangzhou, China, January, 2010 to December, 2012.

Throat samples from 3637 children with LRTI were collected in Guangzhou from January 2010 to December 2012 and 216 samples were identified as influenza A/B positive (5.94%). Among the positive cases, 26 (12.04%) were influenza A H1N1pdm09, 131 (60.65%) were H3N2 and 59 (27.31%) were influenza B (Table 1). Generally, the peak of the detection rates occurred in March each year. After the pandemic period, the detections of influenza A(H1N1)pdm09 reappeared in February and March 2011. Influenza A(H3N2) virus predominated in 2010 and 2012. Influenza B virus was found to co-circulated with H3N2 but fewer cases were identified at the same period of time (Fig 1). The mean age of infection with influenza A H1N1pdm09, H3N2 and influenza B virus were 34.38 months, 32.47 months, and 45.04 months respectively (Table 1). Similar attack rate from all age groups was observed in those children infected with H1N1pdm09 and H3N2. Lower detection rates of influenza B/Yamagata were found in all age groups (less than 10%). However, over 40% of influenza B/Victoria cases were observed in children older than 48 months (Fig 2). We did not find any significant difference in the age distribution of B/Victoria versus B/Yamagata cases (p = 0.50; chi-squared test).

**Table 1:** Clinical presentation and laboratory findings of the children with influenza virus infection.

Clinical Variables	A/H1N1(pdm09)(n = 26)	A/H3N2 (n = 131)	B(n = 59)	P^1^	Clinical Variables	A/H1N1(pdm09)(n = 26)	A/H3N2(n = 131)	B(n = 59)	P^1^
**Males**	16	94	38	0.4304	**Loss of appetite**	7(26.9)	34(26.0)	16(27.1)	0.9838
**Mean age±SD,m**	34.4±34.1	32.5±34.6	45.0±32.5	0.0046	**Stomachache**	1(3.9)	3(2.3)	2(3.4)	ND
**Febrile seizures**	2(7.7)	5(3.8)	3(5.1)	0.6784	**Diarrhea**	1(3.9)	5(3.9)	2(3.4)	ND
**Shivering**	2(7.7)	11(8.4)	9(15.3)	0.3179	**gastrointestinal symptoms**	10(38.5)	53(40.5)	22(37.3)	0.9134
**Fever** ^**2)**^	25(96.2)	116(88.5)	53(89.8)	0.0522	**Crackles**	15(57.7)	59(45.0)	21(35.6)	0.1550
**Max Temp (**°**C)**	39.13±1.04	39.20±0.74	39.22±0.96	ND	**three-concave sign**	1(3.9)	2(1.5)	2(3.4)	ND
**Headache**	2(7.7)	2(1.5)	2(3.4)	0.2053	**Lymphadenopathy**	4(15.4)	39(29.8)	21(35.6)	0.1705
**Myalgia**	0	0	5(8.5)	ND	**Antiadoncus**	14(53.9)	60(45.8)	36(61.0)	0.1445
**Acratia**	0	0	1(1.7)	ND	**Throat herpes**	1(3.9)	2(1.5)	2(3.4)	ND
**Rhinocleisis**	14(53.9)	46(35.1)	20(33.9)	0.1647	**Co-infection of bacteria**	0	6(4.6)	2(3.4%)	ND
**Runny nose**	14(53.9)	71(54.2)	27(45.8)	0.5470	**Co-infection of Mycoplasma Pneumoniae**	10(38.5)	18(13.7)	16(27.1)	0.0054
**Sneezing**	0	10(7.6)	5(8.5)	ND	**Acute bronchitis**	6(23.1)	38(29.0)	21(35.6)	0.4653
**Trachyphonia**	0	2(1.5)	1(1.7)	ND	**Pneumonia**	20(76.9)	93(71.0)	38(64.4)	0.4653
**Cough**	26(100)	129(98.5)	55(93.2)	ND	**Duration of hospitalization**	8.7±5.6	10.0±7.3	9.1±3.5	0.4645
**Sputum production**	5(19.2)	35(26.7)	19(32.2)	0.4657	**Complication**	4(15.4)	34(26.0)	13(22.0)	0.8221
**Wheezing**	6(23.1)	37(28.2)	8(13.6)	0.0877	**Acute benign myositis**	0	0	2(3.4)	ND
**Tachypnea**	2(7.7)	15(11.5)	4(6.8)	0.5797	**Impaired liver function**	1(3.9)	1(0.8)	0	ND
**Vomiting**	4(15.4)	21(16.0)	12(20.3)	0.7425	**Cost median (RMB)**	3765	3821	4254	0.4097

Data are shown as numbers (%) unless otherwise specified.

^1)^ Comparisons were made among A(H1N1)pdm09, seasonal H3N2,and influenza B by using ANOVA for quantitative characteristics and Fisher’s exact or chi-square test for categorical variables, respectively.

^2)^ Denotes the highest body temperature of feverish patients before and on admission to hospital.

ND: Not determined

A higher proportion of the influenza positive cases were identified as pneumonia compared to acute bronchitis in admission diagnoses (H1N1pdm09: 76.9% vs 23.1%; H3N2: 71.0% vs 29.0%; B: 64.4% vs 35.6%). However, there was no significant difference among three groups (p = 0.47) (Table 1). We found that around 37–40% of the patients among the three groups presented gastrointestinal symptoms (H1N1pdm09: 38.5%; H3N2: 40.5%; B: 37.3%) (Table 1). Interestingly, a large proportion of influenza A/B infected hospitalized children were identified with mycoplasma pneumoniae (MP) co-infection. The co-infection rate is higher in the group of influenza A H1N1pdm09 (38.46%) infection compared to the groups of seasonal H3N2 (13.74%,) or influenza B (27.12%) (p<0.05) (Table 1). On the other hand, cases of co-infection with bacteria were rare in our study. The duration of hospitalization in influenza A (H1N1)pdm09, seasonal H3N2 and B group were 8.69±5.55days, 10.00±7.25days and 9.05±3.51days respectively (p = 0.46). All cases were discharged from our hospital and no patients died. We found that the cost of treatment of the children during hospitalization was similar among the three influenza subtypes (3765, 3821, 4254 respectively; p = 0.41).

Chronic respiratory disease appeared to be a risk factor for influenza among the children with LRTI (Table 2). However, no statistically significant differences were observed among the three infection groups with underlying conditions. A high proportion of children with the infection of influenza A(H1N1)pdm09, A(H3N2) and B were living in a secondhand smoking environment (50%, 50%, 46.78% respectively; p = 0.99). The proportion of caesarean was relatively high in each group of our study (46.15%, 44.27% and 44.07% respectively; p = 0.98)

**Table 2:** Personal history of the studied patients with influenza virus infection.

Factors	A/H1N1pdm09 (n = 26)	A/H3N2 (n = 131)	B (n = 59)	P^1)^
**Secondhand smoke**	10(38.5)	45(34.4)	20(33.9)	0.9912
**Caesarean**	12(46.2)	58(44.3)	26(44.1)	0.9823
**≥1 Underlying condition**	6(42.3)	53(40.5)	19(32.2)	0.7917
**Prematurity**	1(3.8)	8(6.1)	7(11.9)	0.2847
**Pulmonary**	4(15.4)	15(11.5)	8(13.6)	0.9722
**Neurologic**	1(3.8)	3(2.3)	1(1.7)	ND
**Cardiac**	0(0)	2(1.5)	2(3.4)	ND
**Renal**	1(3.8)	1(0.8)	0(0)	ND

Data are shown as number (%)

^1)^ Comparisons were made using Fisher’s exact test for categorical variables.

ND: Not determined

## Discussion

We conducted a retrospective surveillance study of influenza A/B infection in hospitalized children with LRTI in Guangzhou city during the post-pandemic period. This study was benefited from the establishment of a sentinel surveillance system for suspected SARS or avian influenza infection cases implemented since 2008. Data from our study showed that three virus subtypes are co-circulating in the community and influenza A (H3N2) was detected more frequently compared to H1N1pdm09 and influenza B among the hospitalized children with LRTI.

The prevalence of the influenza subtypes in hospitalized children with LRTI from this three-year study is consistent with previous findings from the study of outpatients with ILI in the GuangDong province [12]. In early 2010, infection cases of H1N1pdm09 declined dramatically while H3N2 and influenza B were predominant in the population until the end of 2010. The recurrence of H1N1pdm09 was observed during the first half of 2011 and was then replaced by influenza B. In 2012, influenza A H3N2 and influenza B were found to be the major subtypes co-circulating in the population. In general, influenza activity peaked in the spring during the three years. A similar circulation pattern of influenza subtypes in hospitalized children was observed in the central China and Hong Kong in the post-pandemic period when compared to our findings [7,8]. Taken together, our results contribute to a deeper understanding of the prevalence of influenza subtypes in the southern China.

Numerous studies have investigated the age distribution of hospitalized patients who were infected by pandemic and seasonal influenza viruses after the pandemic period [8, 13–16]. Our study focuses on understanding the attack age group of different influenza subtypes in hospitalized children who were suffered from relatively respiratory illness (mainly pneumonia or acute bronchitis) and correlate to the clinical manifestation. A higher mean age was observed from the group of influenza B infection (B: 45.0 months vs H1N1pdm09: 34.4 months vs H3N2: 32.5 months) and a large proportion (>47%) of influenza B virus infected children in our study were older than 48 months. Our findings are consistent to several studies that children with influenza B are usually older than those with influenza A and a close mean age between patients with H1N1pdm09 and seasonal influenza A infection [14, 17–19]. Interestingly, a downward-shift in age distribution was found in the hospitalized children infected by H1N1pdm09, H3N2 or influenza B virus in central China. Different outcomes from the two location of China may associate to the climates, diet or other environmental factors.

Although we have found quite high numbers of patients with gastrointestinal symptoms among the three groups, no difference of any clinical presentation and duration of hospitalization between the infection groups by H1N1pdm09, H3N2 and influenza B viruses was observed. It has been reported that children in Nicaragua had a higher clinical attack rate of H1N1pdm09 compared to the group of seasonal influenza A/B viruses during the pandemic period [20]. The group of H1N1pdm09 showed more symptoms of lower respiratory infection and gastrointestinal symptoms than those who were infected by seasonal influenza viruses. However, an age-matched comparison study from Hong Kong demonstrated that there was no difference in most of the clinical presentation including the gastrointestinal symptoms between the hospitalized children infected by the pandemic and seasonal subtypes [13]. Similar studies have also been done during the post pandemic phase in Athens that the clinical presentation was similar between the children who were infected by the pandemic or seasonal influenza viruses [14]. However, a shorter mean length of hospitalization from the pandemic H1N1 group was observed in the same study. It could be due to the fact that 97% of the patients infected by H1N1pdm09 virus in the study had received oseltamivir while none of the patients in the seasonal cohort received antivirals. On the other hand, there is no routine antiviral therapy such as oseltamivir given to the patients with similar clinical presentation in China. Although study showed that having an underlying neurologic disorder is a risk factor for admission to the ICU with influenza A (H1N1) pdm09 [21], similar association was no found in our study which may due to the limited numbers of related cases.

Interestingly, a high percentage of cases with co-infection of mycoplasma pneumoniae were commonly found in all of our study groups. This finding may be associated with the fact that mycoplasma pneumonia is one of the common pathogens to cause community-acquired respiratory infections and atypical pneumonia [22]. It has been reported that prevalence of this pathogen in children reached to 24.65% in China [23]. However, whether the co-infection of influenza viruses may enhance the pathogenicity during the infection is still needed to be further investigated. While in developed countries, the bacterial co-infection rate reaches up to approximately 28%, only very limited cases from this study were identified as bacterial co-infection [24]. It may due to the different of the clinical practice on frequent use of antibiotics in China.

There are some limitations in this study. Firstly, our study did not conduct in all hospitals in Guangzhou city. Although our hospital is one of the biggest respiratory disease hospitals in Guangzhou, we have no enough data to estimate the total number of influenza virus infected cases among the whole city. Secondly, throat swabs instead of nasopharyngeal swabs were collected and used to detect influenza infection in this study. Our detection may not fully cover all the positive patients who the infection mainly localized at the upper respiratory tract. Thirdly, the patients screening in this study is based on the definition of LRTI but not SARI although there are only slightly differences between them.

In conclusion, higher infection rate of seasonal H3N2 was found in hospitalized children with LRTI than with pdm09 H1N1 and influenza B infection. The severity of the infections was generally mild and no difference in the clinical presentation among the three subtypes was observed. The mycoplasma pneumonia but not bacterial co-infections were commonly found in our cases.

## Supporting Information

S1 Dataset Raw Dataset of the study. (XLSX)
